# Punctate Palmoplantar Keratoderma: A Case Report

**DOI:** 10.7759/cureus.33769

**Published:** 2023-01-14

**Authors:** Ariel Knowles, Maya Adams, Donald A Glass

**Affiliations:** 1 Department of Dermatology, University of Texas Southwestern Medical Center, Dallas, USA; 2 Eugene McDermott Center for Human Growth and Development, University of Texas Southwestern Medical Center, Dallas, USA

**Keywords:** keratoderma, palmoplantar keratoderma, aagab, inheritance, punctate palmoplantar keratoderma

## Abstract

Palmoplantar keratoderma (PPK) is an umbrella term for a group of heterogeneous disorders, acquired or inherited, that are characterized by hyperkeratosis of palmar and/or plantar surfaces. Punctate PPK (PPPK) has been shown to have an autosomal dominant pattern of inheritance. It is linked with two loci on chromosomes 8q24.13-8q24.21 and 15q22-15q24. In type 1 PPPK, also known as Buschke-Fischer-Brauer disease, loss-of-function mutations in either the *AAGAB* or the *COL14A1* genes have been associated with the disorder.^ ^We report here the clinical and genetic features of a patient with findings most consistent with type 1 PPPK.

## Introduction

Palmoplantar keratoderma (PPK) is an umbrella term for a group of heterogeneous disorders, acquired or inherited, that are characterized by hyperkeratosis of palmar and/or plantar surfaces. Three clinical patterns of PPK have been described: diffuse, focal (with extensive hyperkeratosis at points of friction), and punctate [1]. PPKs are usually differentiated based on the morphology and distribution of lesions, histopathologic findings, mode of inheritance, and additional dermatologic and systemic manifestations.

Punctate PPK (PPPK) has been shown to have an autosomal dominant pattern of inheritance. It is linked with two loci on chromosomes 8q24.13-8q24.21 and 15q22-15q24 [2]. In type 1 PPPK, also known as Buschke-Fischer-Brauer disease, loss-of-function mutations in either the *AAGAB* or the *COL14A1* genes have been associated with the disorder [3]. We report here the clinical and genetic features of a patient with findings most consistent with type 1 PPPK.

## Case presentation

**Figure 1:**
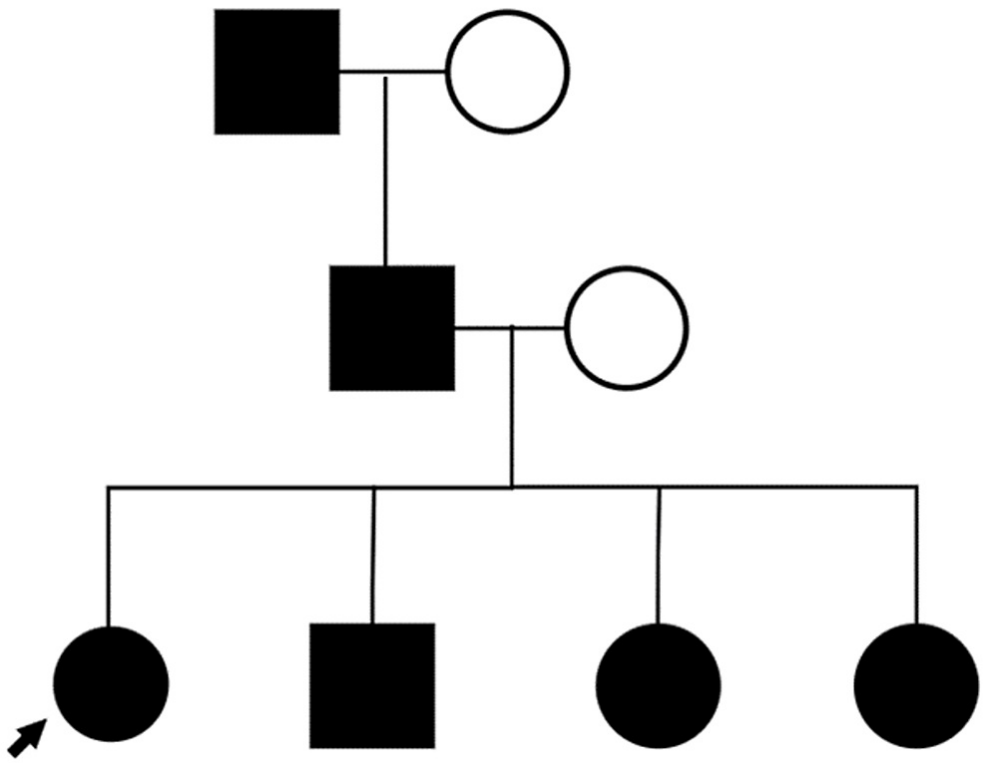
Punctate palmoplantar keratoderma: pattern of inheritance Black indicates affected individuals; arrow indicates proband.

**Figure 2:**
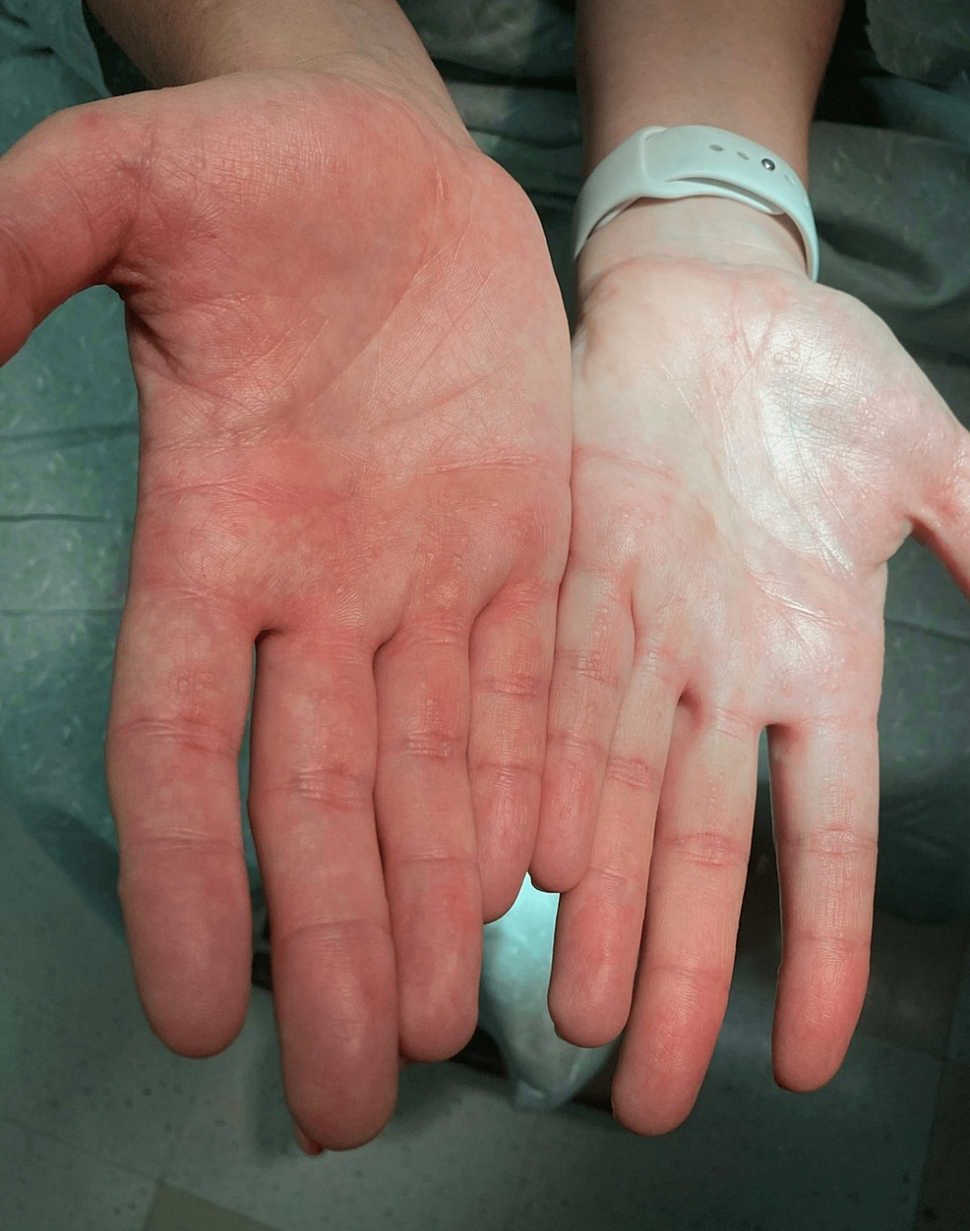
Palmar surface of patient’s hands showing multiple punctate hyperkeratotic papules on the palms and fingers

**Figure 3:**
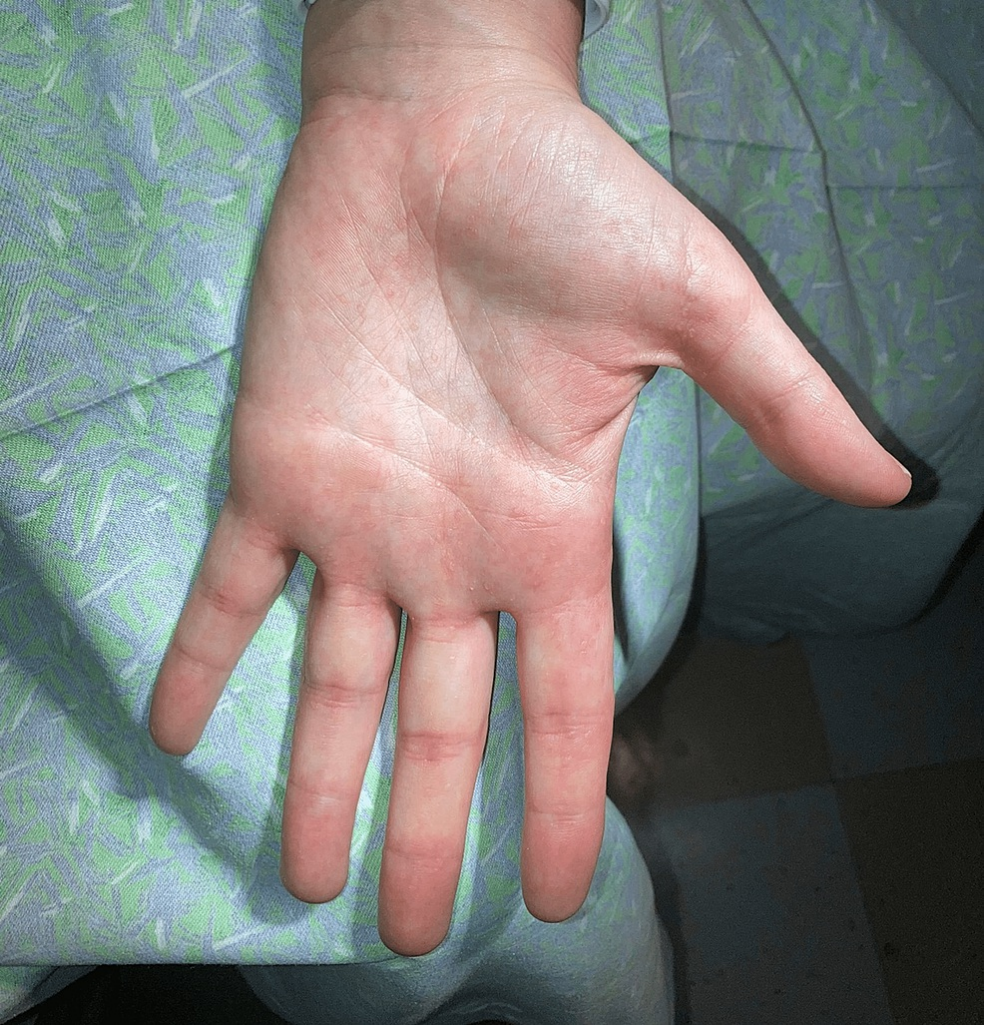
Palmar surface of patient’s left hand showing multiple punctate hyperkeratotic papules on the palms and fingers

A 36-year-old female, with a history of asthma, environmental allergies, and gastroesophageal reflux disease (GERD), presented with a 14-year history of asymptomatic, persistent, and gradually increasing skin lesions on the palms and soles. Her asthma and environmental allergies are well controlled with fluticasone propionate-salmeterol 250-50 µg/dose inhaler one puff inhaled once daily and levocetirizine 5 mg one tablet taken orally once daily. Her GERD is controlled by dietary and lifestyle modifications. Her parents are non-consanguineous. Her paternal grandfather, father, and her three siblings all have similar lesions (Figure 1). Skin examination revealed multiple punctate hyperkeratotic papules, 0.3 to 0.4 cm in size, on the lateral aspects of her fingers, palms, and soles (Figures 2, 3). There were no similar lesions elsewhere on the body. Hair, nails, and mucous membranes were normal. The patient reported using a pumice stone to mechanically debride the papules on her palms and soles.

Genetic sequence analysis and deletion and duplication testing of the *AAGAB* gene performed on a peripheral blood sample revealed a heterozygous non-sense variant [c.370C>T (p.Arg124*)] in exon 4 of the *AAGAB* gene. This variant comprises a known single-nucleotide polymorphism (SNP), rs200564757. This SNP has a minor allele frequency of T = 0.00002509 in an analysis of the Genome Aggregation Database (gnomAD) (https://gnomad.broadinstitute.org/), which combines all populations. To date, the exome analysis has reported this mutation in those of African, European, and Asian ancestral backgrounds. No rare variants were found in the *COL14A1* gene. Based on the above clinicopathological findings, a diagnosis of punctate palmoplantar keratoderma type 1 was made. A biopsy of a lesion was not performed. The patient was reassured and started on 40% urea lotion.

## Discussion

Palmoplantar keratodermas (PPKs) are a family of diseases distinguished by hyperkeratosis of the palms and soles. PPKs can occur in families in different inheritance patterns, including autosomal recessive, autosomal dominant, mitochondrial, or possibly X-linked recessive trait [2]. The interfamilial and intrafamilial severity of the clinical picture shows broad variation [3]. PPKs are clinically classified into three main categories according to the area of the palms and soles affected: diffuse, focal, and punctate [1-2]. The palms and soles undergo a high level of physical stress in everyday use. In mechanically irritated areas, such as these, confluent plaques can be found [3]. PPKs exhibit genetic heterogeneity and have been associated with genes encoding for components of the intermediate filaments, the desmosome, and gap junction channels [2].

The *AAGAB* gene consists of 10 exons with a coding sequence of 945 nucleotides. *AAGAB* has been described to be ubiquitously expressed by array analysis [3]. *AAGAB* is associated with autosomal dominant keratosis palmoplantaris papulosa or punctate palmoplantar keratoderma type 1A (PPKP1A). Giehl et al. [3] showed that mutations in *AAGAB* lead to decreased expression of the AAGAB protein in the skin of those affected by PPKP1A, supporting a role for *AAGAB* in the pathogenesis of PPKP1A. PPKP1A, also known as Buschke-Fischer-Brauer disease, is a rare subtype of palmoplantar keratoderma [2-4]. It is characterized by irregularly distributed hyperkeratotic papules across both palmar and plantar surfaces. Papules might coalesce into larger plaques or acquire a verrucous aspect in areas of greater pressure or friction such as the soles. A typical feature is the worsening of papules after exposure to water [5]. Lesions typically start to develop in late childhood to early adulthood, with a reported age of onset ranging from 12 to 33 years old [6]. Male patients are more commonly affected [7]. It is a rare condition, with a prevalence estimated to be 1.17 per 100,000 persons [8]. To date, over 50 mutations in the *AAGAB* gene encoding for the alpha- and gamma-adaptin-binding protein p34 have been described in the literature [3-5,8-10]. There is no genotype-phenotype correlation [11]. Symptom severity is associated with aging and with environmental factors. There have been reports of an association between PPKP and the development of malignancies, including renal, lung, gastrointestinal, and cutaneous [12]. Treatment options for PPKP1A include moisturizing creams, keratolytics (salicylic acid, lactic acid, urea), topical retinoids, calcipotriene, topical 5-fluorouracil, and oral retinoids, while surgical options include cryosurgery, mechanical debridement, and excision.

## Conclusions

We report a patient with a heterozygous non-sense variant of the *AAGAB* gene leading to punctate palmoplantar keratoderma type 1A (PPKP1A). Her paternal grandfather, father, and her three siblings all have similar punctate hyperkeratotic papules on the palms and soles that appeared in adolescence and increased in severity with age with her older relatives. Prior to her dermatology appointment, the patient managed her palmar and plantar lesions via mechanical debridement. Urea lotion (40%), which was prescribed during her appointment, is expected to aid in the management of her symptoms. Our findings support evidence in the literature that autosomal dominant mutations in *AAGAB* lead to PPKP1A.
